# Revascularization for Coronary Artery Disease and Mitral Regurgitation: A Systematic Review and Meta-analysis

**DOI:** 10.1097/AS9.0000000000000683

**Published:** 2026-06-04

**Authors:** Ryaan EL-Andari, Ashlyn Kruggel, Mitchell Wagner, Evan J. Wiens, Jeevan Nagendran, Jayan Nagendran

**Affiliations:** From the * Division of Cardiac Surgery, Department of Surgery, University of Alberta, Edmonton, AB; † Faculty of Medicine and Dentistry, University of Alberta, Edmonton, AB; ‡ Division of Cardiology, Mazankowski Alberta Heart Institute, University of Alberta, Edmonton, AB.

**Keywords:** coronary artery disease, coronary artery bypass grafting, ischemic mitral regurgitation, percutaneous coronary intervention

## Abstract

**Background:**

Coronary artery disease (CAD) and ischemic mitral regurgitation (IMR) commonly co-exist, yet the optimal intervention for these patients is unclear. Herein, we perform a systematic review and meta-analysis comparing the outcomes of patients with multivessel CAD and IMR undergoing coronary artery bypass grafting (CABG) or percutaneous coronary intervention (PCI).

**Methods:**

PubMed and Embase were systematically searched for articles comparing outcomes of patients with CAD and IMR undergoing revascularization with CABG or PCI, with or without concomitant mitral valve interventions. Thousand two hundred eighty-five studies were identified and 8 were included in this review after full text review. The primary outcome of this study was mortality, and secondary outcomes included myocardial infarction, stroke, heart failure hospitalizations, and residual or recurrent mitral regurgitation.

**Results:**

Pooled analyses identified no significant differences in long-term mortality [odds ratio (OR): 1.13, 95% confidence interval (CI): 0.77–1.68, *P* = 0.53] with considerable heterogeneity between studies. However, following the exclusion of a single outlier, heterogeneity in the pooled analysis of long-term mortality improved and the outcome favored CABG over PCI (OR: 1.39, 95% CI: 1.06–1.83, *P* = 0.02). Rehospitalization for heart failure also favored CABG (OR: 1.49, 95% CI: 1.16–1.92, *P* = 0.002).

**Conclusion:**

This systematic review and meta-analysis suggested lower rates of long-term mortality and heart failure hospitalizations for CABG compared to PCI. CABG remains preferrable for patients with acceptable surgical risk, multivessel CAD, and IMR.

## INTRODUCTION

Mitral valve (MV) regurgitation (MR) involves the back flow of blood across the MV into the left atrium during systole. MR may occur due to abnormalities in the MV apparatus described as primary MR, or in the case of a normal MV such as with a dilated left ventricle or left atrium described as secondary MR.^1^ The co-occurrence of coronary artery disease (CAD) and MR represents a clinically significant intersection of cardiac pathologies, yet their combined incidence and treatment choices remain understudied. The prevalence of CAD is more than 315 million individuals globally in 2022,^2^ and MR affects more than 2% of the population worldwide.^1,3^ MR and CAD often coexist and may carry significant implications for a growing number of patients in the future.^4^

Ischemic mitral regurgitation (IMR) is typically the result ischemia-induced dysfunction of papillary muscles or myocardium (particularly the basal inferior wall). This dysfunction may be acute on the basis of active ischemia; or chronic on the basis of scar, left ventricular (LV) remodelling, MV leaflet tethering or disruption of the MV apparatus.^1,3^ IMR currently affects 1 in 5 individuals who experience a myocardial infarction (MI) and remains closely associated with CAD.^5^ Management of mild-to-moderate MR in patients undergoing coronary artery bypass grafting (CABG) remains most often isolated CABG as evidence suggests no significant benefit of concomitant MV repair (MVr) compared to CABG alone.^6^ In contrast, for patients with severe MR, concomitant CABG with MV replacement (MVR) has been shown to be effective, with reversal of the LV remodeling and improvement in cardiac function.^5,7^ The role of percutaneous coronary intervention (PCI) as an alternative to CABG in the setting of MR is also debated. Following MI, early reperfusion of the vessels using PCI has proven to be important in reducing IMR and promoting LV reverse remodeling, although evidence shows worsened outcomes in individuals presenting with acute IMR.^8^ With a lack of comparative evidence, uncertainty persists regarding whether CABG or PCI offers greater clinical benefit for patients with coexisting MR and CAD.

To date, there is a paucity of comparative analyses between CABG and PCI, with or without concomitant MV intervention for IMR. While PCI allows for early revascularization in the case of acute coronary syndromes, CABG more often allows for complete revascularization in the context of complex CAD and concomitant MV interventions when indicated and the optimal intervention in patients with concomitant CAD and MR is uncertain, especially among the various subgroups. Given these uncertainties, this study aims to systematically evaluate and compare the clinical outcomes of CABG versus PCI in patients with coexisting MR and CAD in order to inform evidence-based treatment strategies. To our knowledge, this systematic review and meta-analysis represents the first comprehensive evaluation of these treatment modalities within a unified analytical framework.

## MATERIALS AND METHODS

### Data Sharing

No raw data is associated with the article as it is a review of the literature.

### Data Sources

**FIGURE 1.**
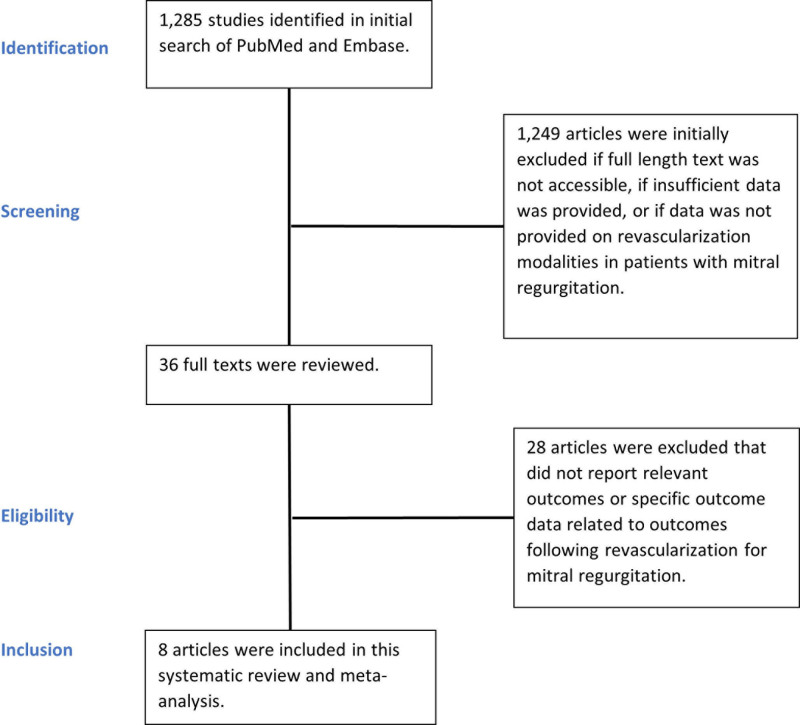
Study flow chart based on the PRISMA guidelines.

This systematic review was conducted in accordance with the Preferred Reporting Items for Systematic reviews and Meta-Analyses (PRISMA, https://links.lww.com/AOSO/A626) guidelines.^9^ A systematic review of the literature was performed utilizing the PubMed and Embase databases (Fig. 1). The search terms used included (coronary artery bypass grafting OR CABG OR revascularization) AND (percutaneous coronary intervention OR PCI OR revascularization) AND (mitral regurgitation, ischemic mitral regurgitation, functional mitral regurgitation). This review was not registered prior to data extraction. Patients and the public were not involved in the design, or conduct, or reporting, or dissemination plans of this research.

### Study Selection

The dates of inclusion for this review were from database inception to March 10, 2025. The inclusion criteria were any investigation comparing CABG and PCI, with or without mitral valve interventions, in patients with concomitant MR of any severity. Included study types were randomized control trials, prospective clinical studies, and retrospective studies. Case reports, reviews, animal studies, abstracts without an associated full text, editorials, studies not available in English, and those that did not report outcomes of interest were excluded.

### Data Extraction

Studies that were identified in the initial literature search were imported into the Covidence Systematic Review Software (Veritas Health Innovation, Melbourne, Australia) for screening. Two authors performed the screening and review of manuscripts. Consensus was required for each manuscript at all steps. In cases of disagreements, the 2 reviewing authors deliberated until consensus was achieved. In the case where consensus was not possible, a third author would act as a tiebreaker.

### Outcomes

The primary outcome of this study was mortality during follow-up. Secondary outcomes included postoperative myocardial infarction, heart failure, readmission to hospital, stroke, required reoperation, and residual or recurrent mitral regurgitation.

### Risk of Bias

A risk of bias assessment was performed for all included studies. The questions used for the risk of bias assessment were derived from the ROBINS-I tool (Cochrane, London, UK) and previous descriptions in publications and included confounding variables, selection bias, performance bias, detection bias, and reporting bias. Funnel plots were used to assess publication bias (Supplemental Figure 1, https://links.lww.com/AOSO/A627). The RevMan 5.4 Software (Cochrane Training, London, UK) was used to create a figure for the risk of bias summary (Supplemental Figure 2, https://links.lww.com/AOSO/A628).

### Statistical Analysis

Data was aggregated and analyzed for the comparison of interventional versus surgical revascularization. Interventional revascularization was defined as PCI with or without concomitant MV interventions. Surgical revascularization was defined as CABG with or without concomitant MV interventions. The outcomes of mortality and heart failure rehospitalization were compared between groups. The degree of difference between studies thought to be due to effect size was assessed using the *I*^2^ statistic. The Chi^2^ statistic was used to measure heterogeneity between studies. In comparisons with insignificant heterogeneity, a fixed effects model was used. In studies with moderate or greater heterogeneity, a random effects model was used. Odds ratio (OR) and 95% confidence intervals (CIs) were calculated using the Mantel–Haenszel method in comparisons with insignificant heterogeneity and the inverse variance method for moderate or greater heterogeneity. The RevMan 5.4 software was used for statistical analyses and to create forest plots for all comparisons. A *P*-value <0.05 was considered to be significant for all comparisons.

## RESULTS

### Overview of Included Studies

Keyword search applied within the aforementioned databases returned 1578 references, with 1249 references having abstracts screened after removal of duplicates. Following abstract review, 36 full texts were chosen for further review, and 8 studies (3 prospective cohort and 5 retrospective) were included in the final analysis (Fig. 1). These studies encompassed a total of 8835 patients: 4916 patients underwent CABG (2993 in isolation and 1921 in conjunction with MVS), and 2614 underwent PCI (38 underwent PCI with concomitant MIVS) (Table 1).

**TABLE 1.** Study Descriptions

Study Name	Study Type	Number of Patients Analyzed	Years Data Collected	Follow-up Period	Study Population	Severity of MR	Exclusion Criteria
Castleberry et al^16^	Retrospective	PCI: 1295 CABG: 1651 CABG + MVR: 243 Total: 3189	1990–2009	Median 5.37 years	Patients undergoing cardiac catheterization with CAD and moderate-severe MR	Moderate–severe MR (2+) PCI 2+: 1006 (77.68) 3+: 244 (18.84) 4+: 44 (3.4) CABG 2+: 1284 (77.77) 3+: 327 (19.81) 4+: 40 (2.42) CABG + MVR 2+: 16 (6.58) 3+: 99 (40.74) 4+: 128 (52.67)	No evidence of significant CAD, prior CABG, history of aortic or pulmonic valvular disease, concomitant valve disease (excluding tricuspid regurgitation), MV prolapse, congenital heart disease, myxomatous or rheumatic MV disease, or endocarditis.
Fan et al^15^	Prospective	PCI: 603 CABG: 587 Total: 1190	2012–2017	4.7 +/−1.8 years	Isolated CABG or PCI	No MR or mild to moderate PCI No-mild MR: 435 (74.1) CABG No-mild MR: 424 (70.3)	Severe MR, concomitant valvular intervention, prior CABG or PCI, acute myocardial infarction within 24h, cardiogenic shock, emergency or life-saving procedure.
Kang et al^12^	Retrospective	PCI: 66 CABG: 119 (+MVR in 68) Total: 185	1996–2008	CABG: median 1613 days (IQR 972–2365) PCI: median 1612 days (IQR 624–2774)	Ischemic MR undergoing CABG (±MVR) vs. PCI	“Significant IMR” – ERO >0.2 cm^2^ Severe MR PCI: 9 (14) CABG: 24 (20)	Organic MV disease (prolapse of mitral leaflets, ruptured chordae, or rupture of papillary Muscles), STEMI and required direct PCI; prior CABG, significant aortic valve disease, required surgical ventricular restoration.
Lin et al^10^	Retrospective	PCI: 57 CABG + MVR: 38 Total: 95	2007–2009	>2 years (median 32 mo.)	Patients undergoing coronary angiography who had acute coronary event and severe MR undergoing revascularization	Severe MR (3+)	>moderate aortic valve disease, lack of complete clinical data.
Mihos et al^13^	Retrospective	PCI + MIVS: 9 CABG + MVS: 15 Total: 24	2009–2014	1 year	PCI + MIVS vs CABG + MVS for IMR and 2 vessel CAD	Moderate-Severe IMR defined as new MR presenting >1 week after diagnosed MI with (I) development of a LV wall motion abnormality; (II) significant CAD in the territory of the wall motion abnormality; and (III) incomplete systolic MV closure with structurally normal MV leaflets MR Grade PCI: 4 (3–4) CABG: 4 (3–4).	Single vessel or three vessel CAD.
Soylu et al^17^	Prospective	PCI: 18 CABG: 17 Total: 35	N/R	3 months	Patients with CAD with moderate-severe MR *Excluded patients who died before follow-up	2–3+ MR MR severity PCI: 2.2 +/−1.4 CABG: 2.4 +/−0.5	Previously known MV disease, 1+ or 4+ MR, history of PCI or CABG, structural abnormality in the MV, technical insufficiency of echocardiography, absence of suggested revascularization, ACS after revascularization, accelerated angina or restenosis.
Trichon et al^14^	Prospective	Medical: 1305 PCI: 537 CABG: 687 CABG + MVS: 228 Total: 2757	1986–2001	Median of 3.2 years	Treatment for ischemic MR with medical therapy, PCI, CABG, or CABG + MVS	≥2+ MR, ≥75% lesion in one or more coronary vessels, and absence of intrinsic mitral valve disease. PCI 2+: 73.6% 3+: 20.7% 4+: 5.8% CABG 2+: 76.0% 3+: 19.9% 4+: 4.1%	Congenital heart disease, primary disease of another cardiac valve, prior CABG or PCI within the previous 12 months, previous valve surgery.
Wang et al^11^	Retrospective	TMVr + PCI: 29 SMVr + CABG: 1331 Propensity matched TMVr + PCI: 29 SMVr + CABG: 133	2016–2018	In-hospital	TMVr + PCI vs SMVr + CABG	Patients with severe IMR scheduled for CABG or PCI with mitral valve repair	Patients younger than 50 years, mitral stenosis, aortic, tricuspid, or pulmonary valve disease, tricuspid valve surgery, pulmonary artery surgery, mitral prolapse, rupture of papillary muscle, rupture of chordae tendinae, and atrial functional mitral regurgitation.

CABG indicates coronary artery bypass grafting; ERO, effective regurgitant orifice; IQR, interquartile range; MR, mitral regurgitation; MVR, mitral valve replacement; MVr, mitral valve repair; MVS, mitral valve surgery; PCI, percutaneous coronary intervention; SMVr, surgical mitral valve repair; TMVr, transcatheter mitral valve repair.

Table 1 provides the details of the included studies. Table 2 compares the reported rates of mortality in individuals with MR undergoing revascularization. Table 3 reviews secondary outcomes such as myocardial infarction, residual MR, readmission for heart failure, etc. The risk of bias assessment identified 6 studies with a low risk of bias, 1 with an uncertain risk of bias, and 1 study with a high risk of bias (Supplemental Figure 2, https://links.lww.com/AOSO/A628).

**TABLE 2.** Rates of Mortality in Patients With Mitral Regurgitation and Coronary Artery Disease Undergoing Revascularization

Study Name	Groups	In-Hospital (%)	30 Days (%)	1 Year (%)	3 Year (%)	5 Year (%)	Longest Follow-up
Castleberry et al^16^	PCI vs CABG PCI vs CABG +MVR	–	–	–	–	–	Adjusted survival ***P* < 0.001** favoring CABG ***P* = 0.0497** favoring CABG
Fan et al^15^	PCI CABG	–	–	–	–	No-mild MR 1.06 (0.73–1.52) 0.78 Moderate MR 2.03 (1.22–3.38) **0.01**	PCI vs CABG: Primary endpoint HR=1.38, 95% Cl: 1.14–1.67, ***P* < 0.001** Cardiovascular death in patients with moderate MR: HR = 1.85, 95% Cl: 1.35–2.55, ***P* < 0.001** Cardiovascular death in patients with no-mild MR: *P* = 0.09 Cardiovascular Death PCI 18.2% CABG 13.6%
Kang et al^12^	PCI CABG, p	–	2 (3.0) 2 (1.7), 0.62	–	–	Actuarial survival: 66 ± 6% 75 ± 5% 0.21	7 years: actuarial survival rate 55 ± 7 60 ± 6, 0.21
Lin et al^10^	PCI CABG + MVR, p	8 (14), 4 (10.5), 0.362	–	–	10 (20.4), 4 (11.8), 0.18	–	
Mihos et al^13^	PCI + MIVS CABG + MVS, p	–	0 0, 1	0 (0) 1 (11), 0.38	–	–	–
Trichon et al^14^	Medical Management	–	–	62.3%	51.8%	41.2%	
	PCI			82.2%	75.0%	68.8%	
	CABG			83.3%	73.7%	64.5%	
	CABG + MVS			79.1%	68.4%	61.6%	
Wang et al^11^	TMVr + PCI SMVr + CABG, p Propensity matched TMVr + PCI SMVr + CABG, p	4 (13.8) 66 (5.0), **0.042** 4 (13.8) 6 (4.5), 0**.034**	–	–	–	–	–

*P*-values bolded if significant.

CABG indicates coronary artery bypass grafting; MR, mitral regurgitation; MVR, mitral valve replacement; MVS, mitral valve surgery; PCI, percutaneous coronary intervention; SMVr, surgical mitral valve repair; TMVr, transcatheter mitral valve repair.

**TABLE 3.** Secondary Outcomes in Patients With Mitral Regurgitation and Coronary Artery Disease Undergoing Revascularization

Study Name	Groups	Myocardial Infarction	Residual MR	Readmission for Heart Failure	Other
Fan et al^15^	PCI CABG, p	–	–	(27.1) (20.2), ***P* < 0.01**	-
Kang et al^12^	PCI CABG CABG + MVr	9 (32.1) 4 (10.5)	Recurrence of MR 2 1	10 (15.2) 11 (9.2)	Improvement of MR 53% 53% 94%
Lin et al^10^	PCI CABG + MVR, p	21 (36.8%) 12 (31.5%), *P* = 0.087	–	14 (28.6), 6 (17.6), *P* = 0.149	Mitral valve reoperation 18 (31.6) 0 (0)
Mihos et al^13^	PCI + MIVS CABG + MVS, p	–	–	–	Stroke 0 0, 1.0
Soylu et al^17^	PCI CABG	–	PCI: Mild – 9 (50) Moderate – 9 (50) CABG: Mild – 8 (47) Moderate – 9 (53)	–	–
Trichon et al^14^	Medical Management PCI CABG CABG + MVS	–	–	–	–
Wang et al^11^	TMVr + PCI SMVr + CABG Propensity matched TMVr + PCI SMVr + CABG	–	–	–	TIA 0 (0) 19 (1.4) 0 (0) 2 (1.5)

*P*-values bolded if significant.

CABG indicates coronary artery bypass grafting; MR, mitral regurgitation; MVR, mitral valve replacement; MVS, mitral valve surgery; PCI, percutaneous coronary intervention; SMVr, surgical mitral valve repair; TMVr, transcatheter mitral valve repair; TIA, transient ischemic attack.

### Mortality Rates Between Coronary Artery Bypass Grafting and Percutaneous Coronary Intervention Over Time

**FIGURE 2.**
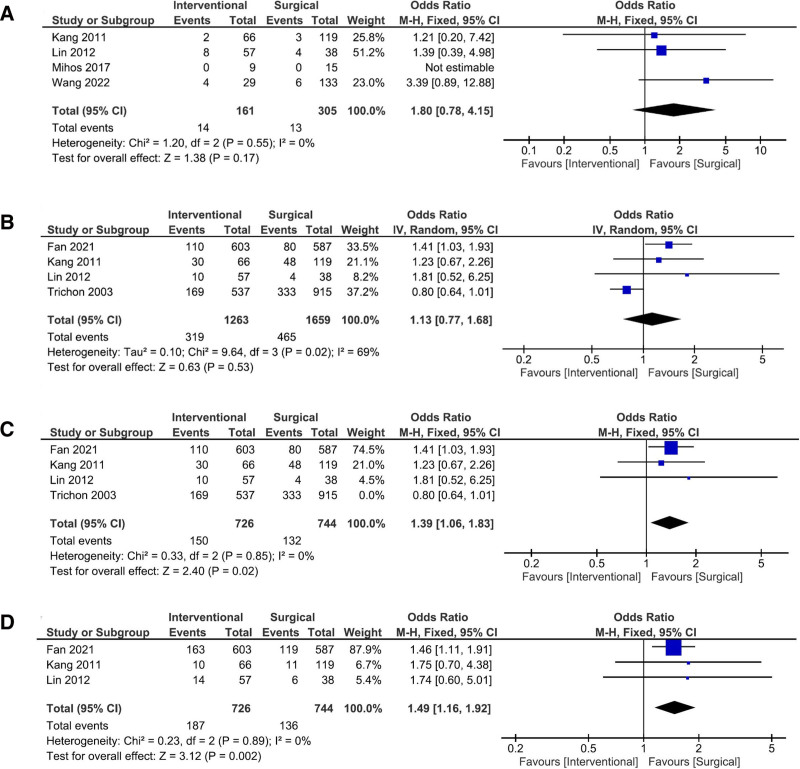
Forest plots for the pooled analyses of 30 day mortality (A), long term mortality (B), long term mortality with the exclusion of one outlier (C), and heart failure rehospitalization (D).

In terms of in-hospital mortality rates, Lin et al reported a 10.5% mortality rate for CABG + MVR vs 14% for PCI *(P* = 0.362), although this did not reach statistical significance.^10^ Wang et al reported a higher mortality rate for propensity-matched patients undergoing PCI + TMVr compared to CABG + SMVr (13.8% vs 4.5%, *P* = 0.034).^11^ One study, Kang et al, reported on 30-day mortality rate, finding no difference between CABG and PCI (1.7% vs 3.0%, *P* = 0.62).^12^ Pooled estimates of 30 day mortality identified no significant difference between interventional or surgical approaches to revascularization (OR: 1.80, 95% CI: 0.78–4.15, *z*-score 1.38, *P* = 0.17) (Fig. 2A).

At 1-year post-operation, Mihos and colleagues reported no significant difference in mortality rates between PCI + MIVS and CABG + MVS^13^; Trichon et al found similarly, with PCI, CABG, and CABG + MVS having minimal difference in survival rates between them at 1-year post-operatively (82.2%, 83.3%, and 79.1%, respectively, no *P*-value reported).^14^ At 3 years postoperation, mortality rates continued to remain similar between groups: for CABG vs PCI, with Lin et al demonstrating a 20.4% vs 11.8% (*P* = 0.18) mortality rate for PCI and CABG, and Trichon et al reported 75.0% vs 73.7% survival, respectively. At 5 years, Fan et al found a higher hazard ratio for cardiovascular death or heart failure hospitalization in PCI vs CABG in patients with moderate MR [2.03 (CI 1.22–3.38), *P* = 0.01] but not in those with none-mild MR [1.06 (CI 0.73–1.52), *P* = 0.78].^15^ Five-year survival rate between CABG vs PCI was reported by Kang et al to be not significantly different (66 ± 6% vs 75 ± 5%, *P* = 0.21), with Trichon et al reporting similarly (64.5% vs 68.8% respectively, no *P*-value reported).

At the longest point of follow-up, most studies showed a benefit of CABG over PCI. Castleberry and colleagues showed a significantly increased median adjusted survival time for CABG or CABG + MVR vs PCI (9.7 vs 8.1 vs 6.8 years, respectively).^16^ Fan et al showed a higher risk of cardiovascular death (18.2% vs 13.6%) and hospitalization for heart failure (27.1% vs 20.2%) for PCI vs CABG.^15^ As a combined endpoint for these measures, there was a higher risk for PCI vs CABG (HR 1.38, *P* < 0.001). In contrast, Kang et al found no significant difference in actuarial survival rate between the two groups (60 ± 6% vs 55 ± 7%, *P* = 0.21).^12^

Mortality at longest follow-up was pooled among 4 studies. There was no significant difference identified in the pooled studies with an OR of 1.13 and 95% CI 0.77–1.68, *z*-score 0.63, *P* = 0.53 (Fig. 2B). There was significant heterogeneity in this comparison with Chi^2^ of 9.64 and *I*^2^ of 69%. Each article was systematically excluded and the removal of a single study, Trichon et al, resulted in a reduction in heterogeneity with a Chi^2^ of 0.33 and *I*^2^ of 0%. With the exclusion of Trichon et al, long-term mortality was reduced in patients that had undergone CABG compared to PCI with an OR of 1.39 and 95% CI that did not cross 1 ranging from 1.06 to 1.83 indicating statistical significance (*z*-score 2.40, *P* = 0.02) (Fig. 2C).

### Secondary Outcomes

Readmission for heart failure was found to be significantly higher in PCI treated patients by Fan et al, with an adjusted HR of 1.45, 95% CI: 1.15–1.84, *P* < 0.01.^15^ Kang et al and Lin et al found numerically higher readmission rates for PCI vs CABG (9.2–17.6% vs 15.2–28.6%).^10,12^ A pooled analysis was performed for reports of readmission for heart failure. Patients who underwent surgical revascularization had a significantly lower rate of readmission to hospital in the pooled analysis with an OR of 1.49, 95% CI: 1.16–1.92, *z*-score 3.12, *P* = 0.002 (Fig. 2D).

Two studies reported on myocardial infarction between PCI and CABG: Kang et al reported a higher rate for PCI (32.1% vs 10.5%, no *P*-value).^12^ Lin et al similarly identified a numerically higher rate of MI in patients who received interventional revascularization, although this did not reach statistical significance (36.8% vs 31.5%, *P* = 0.087).^10^

Residual MR was prevalent in the following studies: Soylu et al found residual moderate MR in 50% of their cohort, with little difference between CABG and PCI.^17^ Though Lin et al did not report on rates of residual MR, re-operation on the MV was undertaken in 31.6% of the PCI group vs none in the CABG group over the course of follow-up.^10^ Kang et al demonstrated improved rates of improvement in MR for patients that underwent concomitant MVr at the time of CABG compared to isolated CABG or PCI.

Other outcomes such as stroke, postoperative atrial fibrillation, and transient ischemic attack were comparable between PCI and CABG groups.^11,13^

## DISCUSSION

IMR is a common occurrence in patients with severe CAD and the optimal treatment of patients with CAD and concomitant MR is often debated. While previous evidence suggests that in patients with CAD and concomitant severe MR, CABG + MVR provides optimal outcomes in terms of surgical approaches, the comparison between surgical and interventional revascularization has received less attention. In this systematic review and meta-analysis, we analyzed the available literature comparing PCI and CABG with or without concomitant MV intervention for patients with severe CAD and MR. This study identified several key findings. Short-term mortality was comparable between PCI and CABG in the individual studies and the pooled analyses. At follow-up greater than 5 years, CABG conferred significant benefits over PCI in terms of survival (Graphical Abstract). Similarly, pooled analyses identified reductions in readmissions to hospital for heart failure in patients treated with CABG compared to PCI. While insufficient data was present for pooled analyses of MI and residual MR, these also favored CABG in the individual studies that reported these outcomes; however, rates of residual MR were not regularly reported and tended to be elevated in studies that performed isolated revascularization, whether surgical or interventional. Other outcomes such as stroke and transient ischemic attack were not significantly different between groups.

The most compelling data available guiding surgical management for patients with IMR has been provided by 2 CTSNet trials in this patient population. Two trials were performed centered around the surgical revascularization and MV intervention for patients with ischemic MR. The CTSNet severe trial compared CABG + MV replacement versus CABG + MV repair for patients with severe IMR.^7^ The study included 251 patients with a 2-year follow-up and identified no significant differences in mortality, although patients who received MV repair were more likely to have recurrent moderate–severe MR and heart failure rehospitalizations than patients who received a replacement. The CTSNet moderate trial included 301 patients with moderate IMR undergoing either isolated CABG or CABG + MV repair and followed patients for 1 year.^6^ This trial found no differences in mortality, a lower rate of residual MR with repair, but a longer hospital length of stay and neurologic events with repair. These trials continue to guide the current management of IMR with patients with severe concomitant MR receiving MV replacement and patients with moderate or less MR receiving isolated revascularization in most cases. These findings are reflected in the guidelines with MV surgery being given a 2a indication in patients undergoing CABG with severe chronic secondary MR.^18^ In the studies included in this review, the highest rates of residual MR were identified in studies that treated patients with isolated revascularization. While the CTSNet trials demonstrated no benefit with mitral valve repair in patients with moderate MR, surgical revascularization was associated with improved survival in this meta-analysis despite increased rates of residual MR in both isolated CABG and PCI. CABG also allows for intervention on the MV when MR is severe. In clinical practice, current evidence suggests that for patients with acceptable surgical risk and multivessel CAD with <severe MR isolated CABG provides the most benefit while in patients with severe MR, combined CABG + MV replacement results in the best outcomes.

Despite the evidence available in the realm of surgical revascularization, direct comparisons between interventional and surgical revascularization modalities in patients with IMR are relatively limited. This study is the first systematic review and meta-analysis to aggregate the available data on this topic and identified significant benefits in terms of survival and heart failure hospitalizations for surgical revascularization. Surgical revascularization allows for a more tailored approach to treatment in this patient population. In patients with severe MR where revascularization alone often does not improve MR, CABG allows for a concomitant MVR which provides benefit to these patients.^7,19^ In patients with moderate or less IMR, MVr is not necessary obviating the need for concomitant transcatheter edge-to-edge repair during PCI or surgical MV repair during CABG. Adequate revascularization will facilitate positive cardiac remodeling in patients with viable myocardium and improvement in MR in a subset of patients without a primary MV problem.^20,21^

Future studies on this investigation should aim to directly compare isolated revascularization and revascularization with MV interventions. With heterogeneous presentations of patients with IMR in terms of CAD severity, urgency of presentation, MR severity, and MV morphology, and several potential interventional options, the optimal interventions for this patient population likely include a combination of CABG, CABG + MVS, PCI, and PCI with transcatheter edge-to-edge repair. Future detailed comparisons and granular data will be required to determine the optimal intervention for each subset of patients. Based on the available evidence, in patients with severe multivessel CAD and IMR, surgical revascularization with CABG with or without surgical mitral valve replacement remains preferable for this patient population in appropriately selected patients. PCI will continue to play a crucial role in managing this patient population, especially in patients presenting with STEMI requiring early revascularization, those with high surgical risk or short term life expectancy that may not confer the long-term benefits of CABG, and patients with non-complex CAD.

### Limitations

The main limitation of this systematic review is the relatively limited data available for a subset of secondary outcomes such as myocardial infarction and stroke which precluded aggregation of data and pooled analysis. There was also some heterogeneity between studies in terms of intervention with some studies pooling surgical interventions (CABG and CABG + MVS) while others reported them separately or only isolated revascularization. The findings regarding the data collected and pooled in this review assume exclusion restriction and independence between the patients, interventions and outcomes which cannot be confirmed or disproven in this setting. The absence of randomized control trials in this field limits the definitive conclusions that can be drawn regarding this comparison and the addition of randomized control trials would help to address the aforementioned limitations. While aggregation of this data replicates real-world interventions performed for these patients, direct comparisons of isolated CABG or PCI and revascularization with MV interventions are warranted.

## CONCLUSIONS

This systematic review and meta-analysis compared interventional and surgical approaches to revascularization and mitral valve interventions among patients with severe CAD and ischemic MR. This review identified lower rates of long-term mortality and heart failure hospitalizations for patients who underwent surgical revascularization compared to patients who underwent percutaneous revascularization. CABG for severe CAD with IMR allows for tailoring of the procedure, allowing for complete and durable revascularization for multivessel CAD along with the option for MV replacement in cases of severe MR. While CABG remains preferable for patients with acceptable surgical risk, multivessel CAD, and IMR, PCI remains an indispensable tool for managing this patient population where CABG is not indicated or feasible and future research is needed to identify patient populations that may benefit more from one intervention or the other.

## ACKNOWLEDGEMENTS

We would like to thank Dr. Yongzhe Hong for his review of the statistical analysis of this manuscript.

## AUTHOR CONTRIBUTION

R.El.-A.: study design, data collection, data analysis, data interpretation, writing of the manuscript, approval of final manuscript; A.K.: study design, data collection, data analysis, data interpretation, writing of the manuscript, approval of final manuscript; M.W.: study design, data collection, data analysis, data interpretation, writing of the manuscript, approval of final manuscript; E.W.: study design, data interpretation, proofreading and revisions, approval of final manuscript; J.N.: study design, data interpretation, proofreading and revisions, approval of final manuscript; J.N.: study design, data interpretation, proofreading and revisions, approval of final manuscript.

## Supplementary Material

## References

[1] Chehab O Roberts-Thomson R Ng Yin Ling C. Secondary mitral regurgitation: pathophysiology, proportionality and prognosis. Heart. 2020;106:716–723. 32054671 10.1136/heartjnl-2019-316238

[2] Stark B Johnson C Roth GA. Global prevalence of coronary artery disease: an update from the global burden of disease study. J Am Coll Cardiol. 2024;83:2320.

[3] Enriquez-Sarano M Akins CW Vahanian A. Mitral regurgitation. Lancet. 2009;373:1382–1394. 19356795 10.1016/S0140-6736(09)60692-9

[4] Lapar DJ Acker MA Gelijns AC. Repair or replace for severe ischemic mitral regurgitation: prospective randomized multicenter data Keynote Lecture Series. Ann Cardiothorac Surg. 2015;4:411–416. 26539344 10.3978/j.issn.2225-319X.2015.04.11 PMC4598462

[5] Varma PK Krishna N Jose RL. Ischemic mitral regurgitation. Ann Card Anaesth. 2017;20:432–439. 28994679 10.4103/aca.ACA_58_17 PMC5661313

[6] Smith PK Puskas JD Ascheim DD; Cardiothoracic Surgical Trials Network Investigators. Surgical treatment of moderate ischemic mitral regurgitation. N Engl J Med. 2014;371:2178–2188. 25405390 10.1056/NEJMoa1410490 PMC4303577

[7] Goldstein D Moskowitz AJ Gelijns AC; CTSN. Two-year outcomes of surgical treatment of severe ischemic mitral regurgitation. N Engl J Med. 2016;374:344–353. 26550689 10.1056/NEJMoa1512913 PMC4908819

[8] Nishino S Watanabe N Kimura T. The course of ischemic mitral regurgitation in acute myocardial infarction after primary percutaneous coronary intervention. Circ Cardiovasc Imaging. 2016;9:e004841. 27516478 10.1161/CIRCIMAGING.116.004841

[9] Page MJ McKenzie JE Bossuyt PM. The PRISMA 2020 statement: an updated guideline for reporting systematic reviews. BMJ. 2021;372:n71. 33782057 10.1136/bmj.n71 PMC8005924

[10] Lin KL Hsiao SH Wu CJ. Treatment strategies for acute coronary syndrome with severe mitral regurgitation and their effects on short- and long-term prognosis. Am J Cardiol. 2012;110:800–806. 22640972 10.1016/j.amjcard.2012.05.010

[11] Wang X Ma Y Liu J. Outcomes of transcatheter edge-to-edge mitral valve repair with percutaneous coronary intervention vs. surgical mitral valve repair with coronary artery bypass grafting. Front Cardiovasc Med. 2022;9:953875. 36620639 10.3389/fcvm.2022.953875 PMC9810628

[12] Kang DH Sun BJ Kim DH. Percutaneous versus surgical revascularization in patients with ischemic mitral regurgitation. Circulation. 2011;124:S156–S162. 21911806 10.1161/CIRCULATIONAHA.110.011254

[13] Mihos CG Xydas S Williams RF. Staged percutaneous coronary intervention followed by minimally invasive mitral valve surgery versus combined coronary artery bypass graft and mitral valve surgery for two-vessel coronary artery disease and moderate to severe ischemic mitral regurgitation. J Thorac Dis. 2017;9:S563–S568. 28740708 10.21037/jtd.2017.04.17 PMC5505937

[14] Trichon BH Glower DD Shaw LK. Survival after coronary revascularization, with and without mitral valve surgery, in patients with ischemic mitral regurgitation. Circulation. 2003;108:II103–II110. 12970217 10.1161/01.cir.0000087656.10829.df

[15] Fan Q Liu J Xu Y. Real-world outcomes of revascularization strategies in patients with left ventricular dysfunction and three-vessel coronary disease stratified by mitral regurgitation. Front Cardiovasc Med. 2021;8:675722. 34250038 10.3389/fcvm.2021.675722 PMC8265779

[16] Castleberry AW Williams JB Daneshmand MA. Surgical revascularization is associated with maximal survival in patients with ischemic mitral regurgitation: a 20-year experience. Circulation. 2014;129:2547–2556. 24744275 10.1161/CIRCULATIONAHA.113.005223 PMC4142433

[17] Soylu K Kocakavak C Demircan S. The effect of the revascularization strategies on the severity of ischemic moderate mitral regurgitation. Eastern J Med. 2013;18:16–22.

[18] Otto CM Nishimura RA Bonow RO; Writing Committee Members. 2020 ACC/AHA Guideline for the management of patients with valvular heart disease: a report of the American College of Cardiology/American Heart Association Joint Committee on Clinical Practice Guidelines. J Am Coll Cardiol. 2021;77:e25–e197. 33342586 10.1016/j.jacc.2020.11.018

[19] Yamazaki S Numata S Yaku H. Surgical intervention for ischemic mitral regurgitation: how can we achieve better outcomes? Surg Today. 2020;50:540–550. 31147764 10.1007/s00595-019-01823-8

[20] Penicka M Linkova H Lang O. Predictors of improvement of unrepaired moderate ischemic mitral regurgitation in patients undergoing elective isolated coronary artery bypass graft surgery. Circulation. 2009;120:1474–1481. 19786637 10.1161/CIRCULATIONAHA.108.842104

[21] Campwala SZ Bansal RC Wang N. Mitral regurgitation progression following isolated coronary artery bypass surgery: frequency, risk factors, and potential prevention strategies. Eur J Cardiothorac Surg. 2006;29:348–353. 16442297 10.1016/j.ejcts.2005.12.007

