# Supplementary figures and images for: Revascularization for Coronary Artery Disease and Mitral Regurgitation: A Systematic Review and Meta-analysis
Source: Ann Surg Open. 2026 Jun 4;7(2):e683. doi: 10.1097/AS9.0000000000000683 (PMC13290160; doi:10.1097/AS9.0000000000000683)

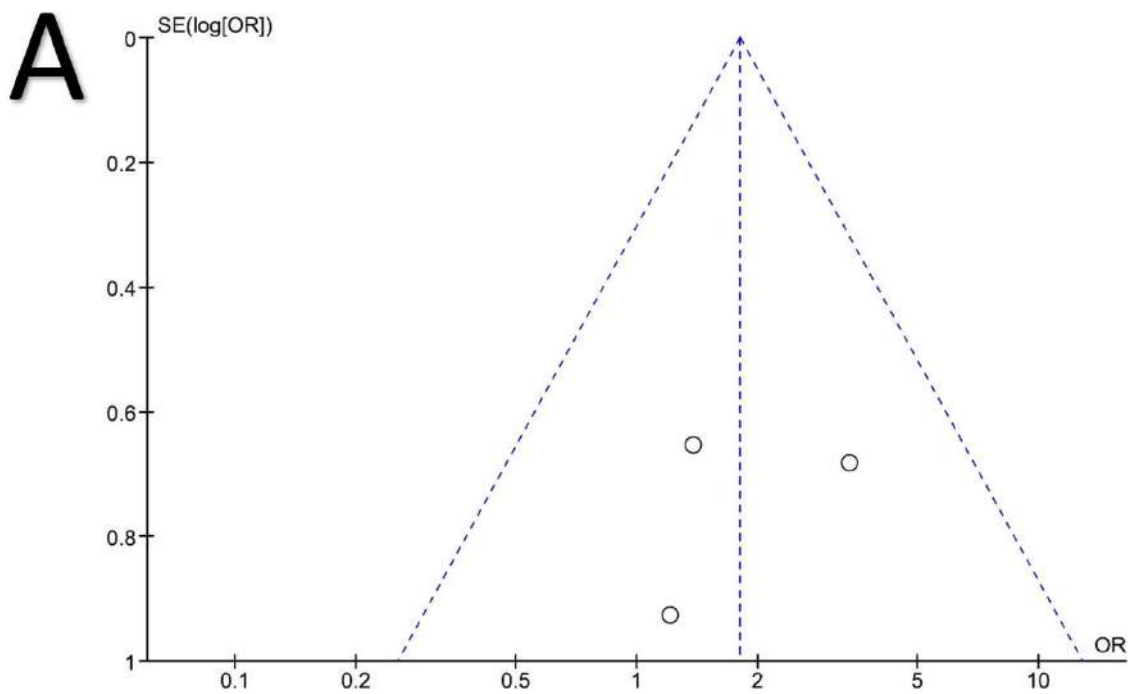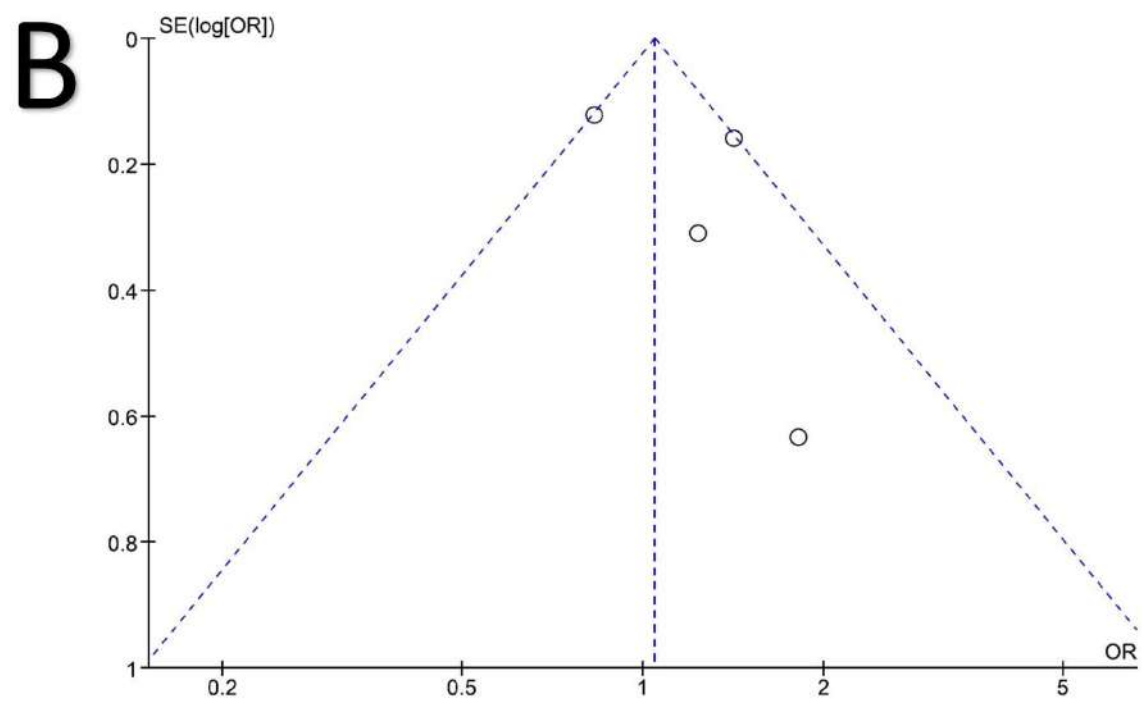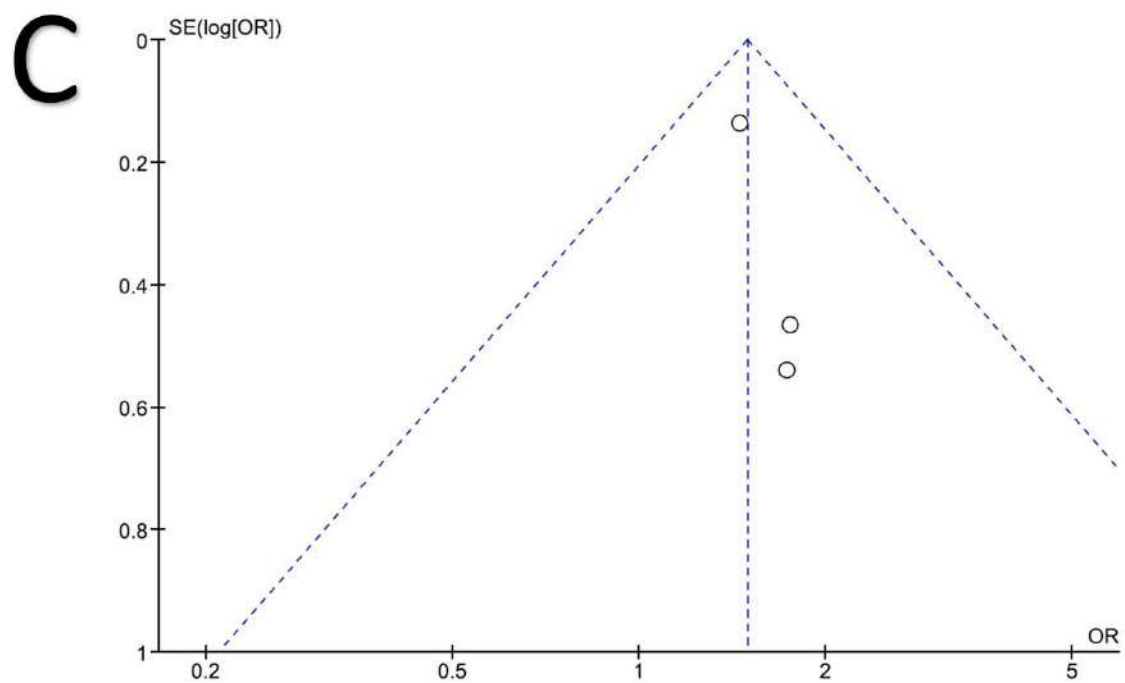

Supplement: Supplementary file 2 [file as9-7-e683-s002.pdf]
