# Supplementary material for: Revascularization for Coronary Artery Disease and Mitral Regurgitation: A Systematic Review and Meta-analysis
Source: Ann Surg Open. 2026 Jun 4;7(2):e683. doi: 10.1097/AS9.0000000000000683 (PMC13290160; doi:10.1097/AS9.0000000000000683)
Supplement: Supplementary file 3 [file as9-7-e683-s003.pdf]

	Recruitment Strategy	Inclusion and Exclusion Criteria	Group Selection	Sample Size	Matching	Statistical Methods	Classification of Outcomes	Missing Important Outcomes	Follow-up Time	Incomplete Follow-up	Missing Data	Confounding Variables	Overall Assessment of Bias
Castleberry 2014	+	+	+	+	+	+	+	−	+	+	+	+	+
Fan 2021	+	+	+	+	+	+	+	+	+	+	+	+	+
Kang 2011	+	+	?	?	+	+	+	+	+	+	+	?	+
Lin 2012	+	+	+	+	+	+	+	+	?	+	?	+	+
Mihos 2017	+	+	+	−	−	−	−	+	?	+	+	+	?
Soylu 2013	?	+	+	−	−	?	+	+	−	+	−	−	−
Trichon 2003	+	+	+	+	+	+	?	+	+	+	+	−	+
Wang 2022	+	+	+	+	+	+	+	+	−	+	+	+	+
